# Design and Finite Element Evaluation of a Customized 3D-Printed Wrist-to-Finger Orthosis Targeting Carpal Tunnel Pressure Reduction

**DOI:** 10.3390/polym18202521

**Published:** 2026-10-15

**Authors:** Muhammad Abbas, Niyonkuru Benjamin, Addisu Negash Ali, Kashif Azher, Suhail Hyder Vattathurvalappil, Aamer Nazir

**Affiliations:** 1 Department of Mechanical Engineering, King Fahd University of Petroleum & Minerals, Dhahran 31261, Saudi Arabia; 2 Faculty of Mechanical and Industrial Engineering, Bahir Dar Institute of Technology, Bahir Dar University, Bahir Dar P.O. Box 26, Ethiopia; 3 Department of Machining, Assembly and Engineering Metrology, Faculty of Mechanical Engineering, VŠB-Technical University of Ostrava, 17. Listopadu 2172/15, 708 00 Ostrava, Czech Republic; 4 Interdisciplinary Research Center for Advanced Materials, King Fahd University of Petroleum & Minerals, Dhahran 31261, Saudi Arabia; 5 Department of Aerospace Engineering, King Fahd University of Petroleum and Minerals, Dhahran 31261, Saudi Arabia

**Keywords:** carpal tunnel syndrome, wrist–hand orthosis, finite element analysis, multi-material additive manufacturing, customized design, biomechanics

## Abstract

Carpal tunnel syndrome (CTS) is a common condition caused by increased carpal tunnel pressure and compression of the median nerve. Wrist–hand orthosis is widely used as a nonsurgical treatment to maintain the wrist in a near-neutral position and reduce pressure within the carpal tunnel. However, conventional orthosis often suffers from poor comfort, inadequate ventilation, localized pressure, and low patient compliance. This study aimed to design and evaluate a customized, multi-material 3D-printed wrist-to-finger orthosis that incorporated individual finger supports, perforated regions, and a volar clearance zone intended to avoid direct shell contact over the carpal tunnel region. A customized proof-of-concept orthosis was designed using a dual-material structure consisting of a rigid polylactic acid (PLA) outer shell and a flexible thermoplastic polyurethane (TPU) inner liner. The TPU liner was incorporated at skin contact regions to improve comfort, while the PLA shell provided structural support. Perforated lattice patterns were integrated to enhance ventilation and reduce weight, and a dedicated clearance zone was created on the volar side of the wrist to minimize direct pressure on the carpal tunnel area. Finite element analysis (FEA) was conducted under three loading conditions to evaluate stress distribution and deformation. The design refinement reduced the calculated mass from 258.27 to 205.36 g, corresponding to a 20.5% reduction. The maximum sampled von Mises stresses on the final PLA shell were 1.04 MPa on the upper surface and 0.99 MPa on the lower surface. Flexural testing demonstrated that the orthosis possessed adequate stiffness and maintained structural integrity under applied loads. The developed customized proof-of-concept orthosis demonstrates the feasibility of combining multi-material fused filament fabrication (FFF), personalized design, and FEA to produce a lightweight, breathable, comfortable, and mechanically reliable device for CTS management. The incorporation of a TPU liner and a carpal tunnel clearance zone has the potential to improve user comfort while maintaining the mechanical support required for effective treatment.

## 1. Introduction

The upper limbs play an important role in performing daily activities. Due to different types of accidents, some people may lose an upper limb or experience limited functioning of the arm and hand. As a result, basic activities such as eating, dressing, and personal care can become difficult. Assistive devices can help these individuals regain some of their lost abilities and improve their independence. Upper limb orthotic devices are commonly used in rehabilitation because they help prevent or correct deformities, support and stabilize joints, assist weakened muscles, transfer muscle forces, and improve overall limb function [1]. These devices help improve or restore the function of the affected limb. For this reason, orthosis is widely used in patients with neurological conditions involving the brain, spinal cord, or peripheral nerves. Orthosis is used to manage musculoskeletal and peripheral nerve conditions, including fractures, sprains, joint disorders, tendon injuries, and peripheral nerve injuries [1,2].

Many prefabricated commercial wrist splints are supplied in a limited range of sizes and may not adequately conform to individual anatomy. Poor fit can produce localized pressure and friction, impair thermal comfort, and reduce adherence. Carpal tunnel syndrome (CTS) is a compression neuropathy of the median nerve at the wrist. Carpal tunnel pressure varies with wrist posture and generally increases when the wrist moves substantially away from the neutral position [3]. Studies have reported that the lowest carpal tunnel pressure occurs when the wrist is in a neutral or slightly extended position. This finding supports the use of splints designed to keep the wrist at these angles. Maintaining this posture can help reduce pressure around the wrist area and provide relief from the symptoms associated with carpal tunnel syndrome [4]. Similar conditions can also occur in Acute Compartment Syndrome (ACS), where pressure increases inside a closed muscle compartment, such as in the forearm or wrist. When a cast or a tight bandage limits the natural swelling of the tissues, the internal pressure can increase. This pressure may compress blood vessels and reduce blood flow to the affected area. As a result, tissue damage and nerve-related problems may occur, including conditions such as carpal tunnel syndrome (CTS) and ACS [5]. These findings provide a biomechanical rationale for maintaining the wrist in a near-neutral position; however, they do not independently establish clinical symptom relief. Patient-specific workflows that combine three-dimensional scanning, digital modeling, and additive manufacturing can improve anatomical conformity and enable openwork structures and localized relief features.

Additive manufacturing (AM) provides the ability to produce customized medical devices. In recent years, the use of 3D printing in the orthotics field has increased significantly. This trend is expected to continue because of the rapid development of new materials and the decreasing cost of many additive manufacturing technologies [6,7]. Rigid orthosis often requires a balance between providing sufficient immobilization and maintaining user comfort. Multi-material additive manufacturing (MMAM) offers the possibility of using different material properties within the same device. This allows stiffer regions to provide support and posture control, while softer regions improve comfort in areas that are in contact with the skin. In addition, additive manufacturing improves traditional wrist splints by using accurate anatomical data to produce customized orthosis with a better fit and improved ventilation [8]. Although this technology provides greater design flexibility and improved user comfort, clinical supervision is still important. Proper medical guidance is needed to reduce the potential health risks that may result from self-designed or self-fabricated devices [9]. A recent study on 3D-printed wrist–hand orthosis examined the use of PLA material to improve user comfort while still providing the level of immobilization required for effective treatment [10]. Another study developed a customized wrist cast made from PLA and TPU materials. The design used topology optimization and was manufactured using FFF. In addition, an artificial neural network (ANN) optimized with a genetic algorithm (GA) was used to improve the design. The developed cast showed improvements in mechanical strength, ventilation, and user comfort [11]. Górski et al. [12] presented an automated method for generating individualized openwork wrist–hand orthosis and evaluated their fit and strength. These studies have established the use of customization, additive manufacturing, ventilation features, numerical analysis, and mechanical testing in wrist–hand orthosis.

The contribution of the present work is therefore the combination of a wrist-to-finger architecture with individual finger interfaces, a dedicated volar relief zone, compliant TPU contact regions, and manual stress-guided regional lightweighting within one engineering prototype; it does not claim to introduce the first customized, ventilated, or multi-material hand orthosis. Accordingly, this study designed and fabricated a geometry-adapted wrist-to-finger orthosis with rigid PLA components and compliant TPU interfaces. FEA was used as a structural screening tool to examine load paths and guide regional material removal, and device-level bending was used to characterize preliminary flexural behavior. Because the starting geometry was a representative open-source model and no direct pressure or clinical measurements were performed, the device is presented as an anatomy-based engineering proof of concept rather than a validated patient-specific treatment.

## 2. Materials and Methods

### 2.1. Design Requirements

The design requirements were wrist stabilization near a neutral position, a volar clearance region, reduced component mass, perforated regions, support for finger positioning, visual access to selected skin regions, and a fastening system that permitted donning and doffing. The volar clearance was intended to avoid direct shell contact over the anatomical carpal tunnel region while maintaining wrist alignment [13]. This design feature was not evaluated as a measure of skin interface pressure or intratunnel pressure. Maintaining this position is essential for reducing stress on the median nerve and improving patient comfort during prolonged use. To enhance usability and comfort, the orthosis should include ventilation features through patterned perforations distributed across the structure [14]. These perforations promote airflow, improve breathability, and reduce the accumulation of heat and moisture within the device, which can otherwise lead to skin irritation during extended wear [15]. The orthosis should also be designed to be a lightweight structure, achieved by strategically incorporating perforations that reduce overall mass while maintaining structural reinforcement features to preserve stiffness and mechanical support [16]. In addition to wrist stabilization, the device should extend support to the fingers through modular finger supports to improve control of finger posture during wrist motion, as variations in finger position and applied force have been shown to significantly influence carpal tunnel pressure and median nerve biomechanics [17].

For safety and monitoring purposes, the design should include inspection or access windows, particularly over superficial regions such as veins and sensitive skin regions. These windows allow visual inspection of the skin, reduce localized pressure over superficial tissues, and help prevent circulation issues or skin irritation. Furthermore, the orthosis should be manufactured using biocompatible and skin-friendly materials to ensure comfort and safety during prolonged contact with the skin, as outlined in the ISO 10993 standards [18]. Such materials should be lightweight, non-toxic, and hypoallergenic, reducing the risk of skin irritation while providing sufficient flexibility and durability for daily use. Finally, the design should incorporate a simple and user-friendly fastening mechanism, allowing the wearer to easily put on and remove the orthosis independently without assistance. Adjustable straps or similar closure systems can provide secure fixation while enabling quick donning and doffing, thereby improving usability and patient compliance. Making an adjustable fastening mechanism is important, as it will improve the patient’s comfort by allowing them to put on and remove the orthosis themselves. Also, if temporary swelling occurs in the region, the orthosis can be tightened and loosened easily, which can help reduce the risk of many medical issues like gangrene [19].

### 2.2. Design Process and FEA-Guided Structural Refinement

**Figure 1:**
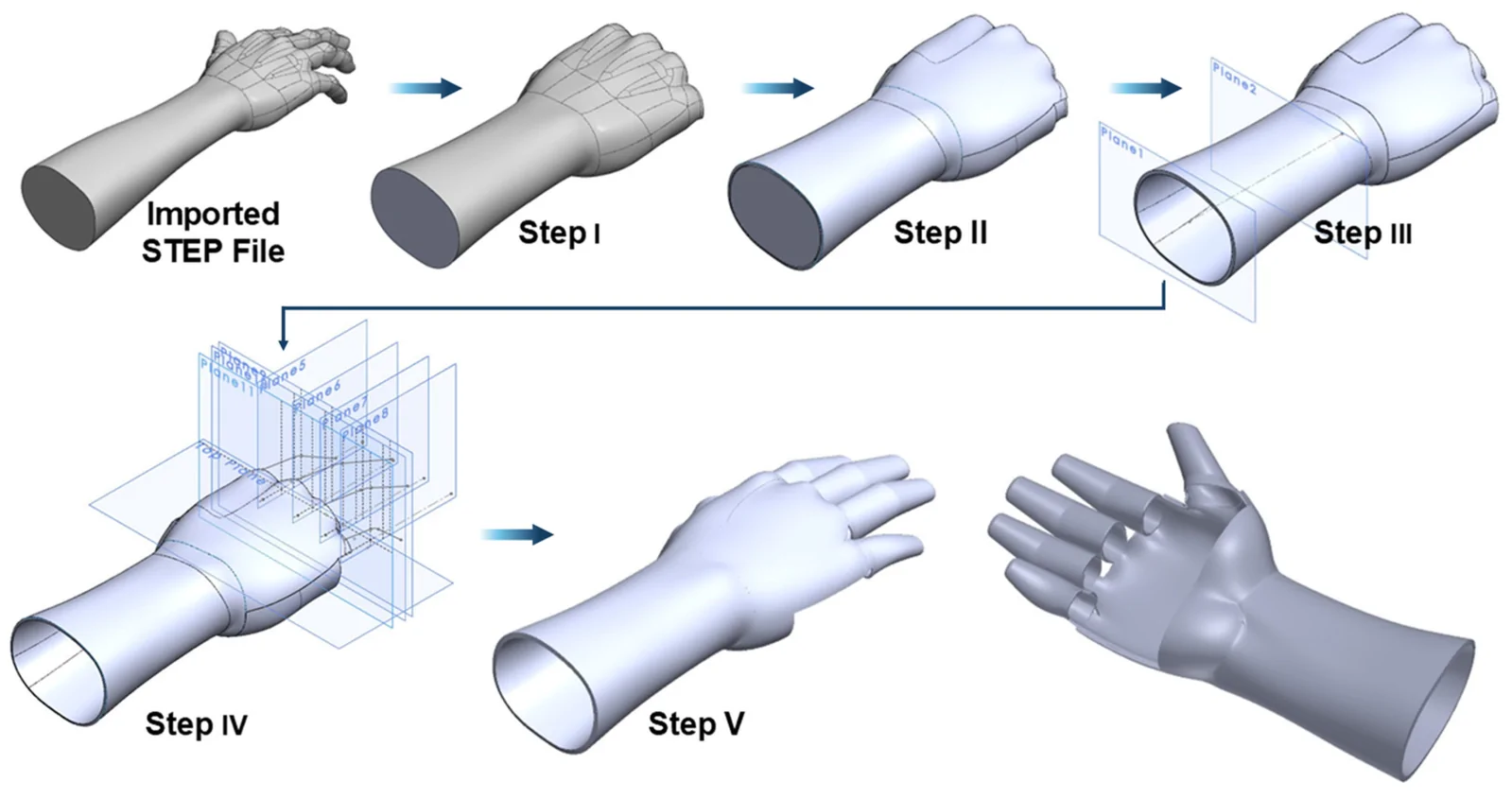
Workflow for developing the wrist-to-finger orthosis geometry in SOLIDWORKS. The process starts with an imported STEP model of the 3D-scanned human arm, followed by resizing and removing the original fingers (Step I). In Step II, surface smoothing and offsetting are performed to create the inner layer, which is then converted into a solid using the thicken feature. Step III shows the creation of a hollow wearable structure using Boolean subtraction. In Step IV, new fingers are built using reference planes. Finally, Step V presents the completed orthosis geometry after applying the open palm cut and outer layer design.

The wrist-to-finger orthosis geometry was created in SOLIDWORKS (2024, Dassault Systèmes, Vélizy-Villacoublay, France) using an open-source 3D-scanned STEP model downloaded from GrabCAD [20]. This solid model was selected to capture the anatomical surface morphology of the human arm. Using SOLIDWORKS surface features, it was scaled and modified to fit an individual whose arm morphology closely matched that of the imported model. Figure 1 (I–V) illustrates the design workflow, from the imported model to the final CAD geometry used in this study. In Step (I), the model was scaled to the target dimensions, and the original fingers were removed because their length and flexion angles were not appropriate for the selected individual. (II) The dorsal hand was smoothed using surface editing features (Delete and Fill) to reduce fragmented faces, enabling reliable offsetting. Within the same step, an offset surface of 2.0 mm was applied to define the intended inner layer, followed by employing a “thicken feature” to convert the offset surface into a solid shell with controlled thickness. (III) A wearable hollow structure was created by Boolean subtraction of the imported body from the thickened offset solid. (IV) New fingers were built parametrically by defining individual finger reference planes, sketching a finger trajectory, creating three spaced cross-sectional profiles, and generating each finger using a path-guided loft. The structure was finalized by creating an open palm through a planar cut using the top plane established in Step IV, after which the outer layer was designed using the same surface-based workflow.

**Figure 2:**
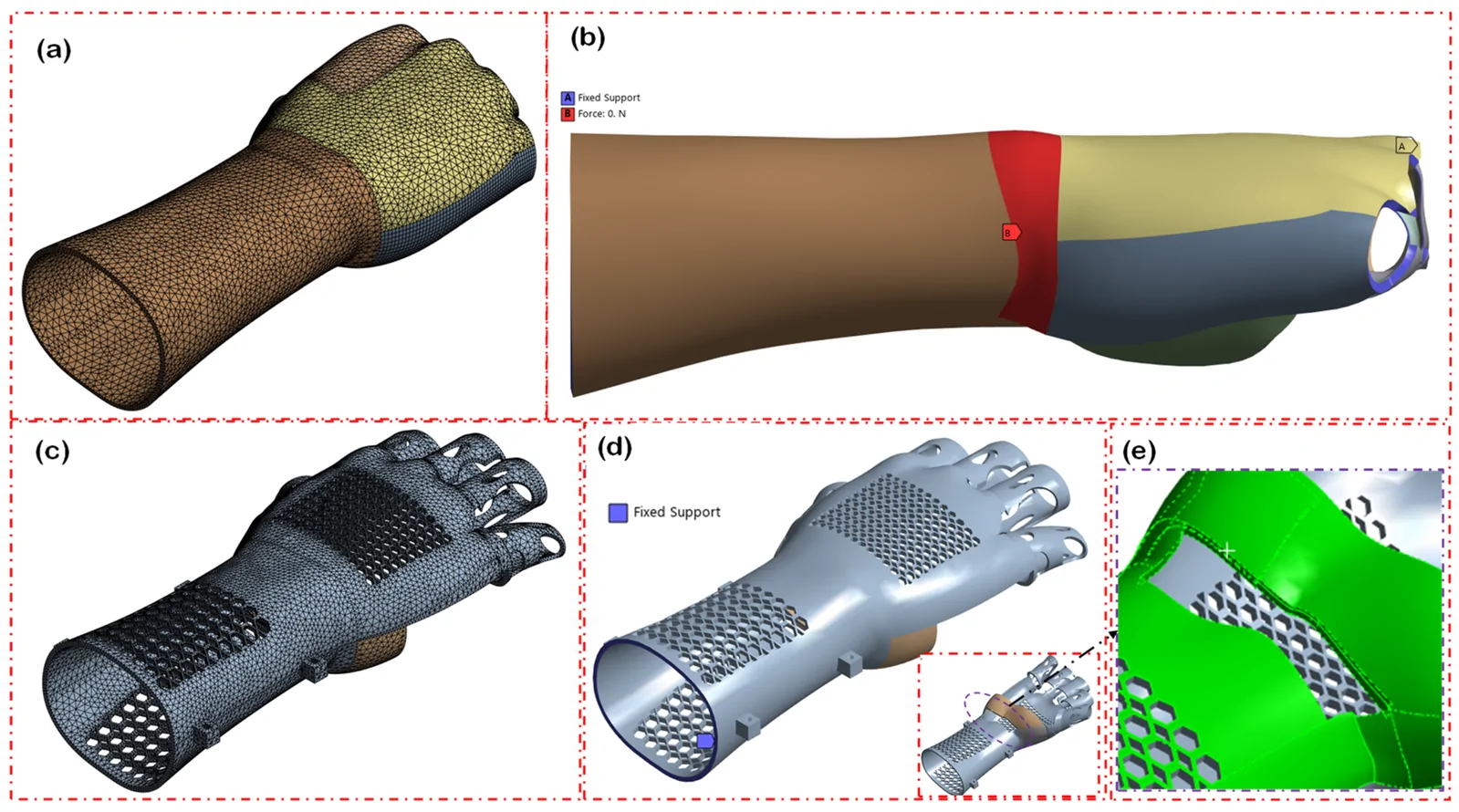
(**a**) Geometry and structural screening setup: (**a**) representative forearm–hand geometry and finite element mesh, (**b**) constraints applied in the idealized structural model, (**c**) mesh of the perforated orthosis positioned on the representative geometry, (**d**) CAD model showing the finger supports and perforated regions, and (**e**) enlarged view of the volar relief and inspection region. The figure does not show measured or clinically validated pressure reduction.

The geometry of the arm for the corresponding orthosis model was imported into ANSYS Static Structural 2024R2 and discretized into finite element meshes (Figure 2a,c). A material model reports the exact inputs used in ANSYS Workbench engineering data, and PLA was modeled using a linear elastic–plastic model, which was validated in our previous study [21], with a density of 1250 kg/m^3^, an elastic modulus of 1.5 GPa, a Poisson’s ratio of 0.35, and yield strength of 42 MPa. TPU was modeled using the two-parameter Mooney–Rivlin model [22], with C01 = 4.39 MPa and C10 = 1.15 MPa. The PLA model did not reproduce build direction-dependent orthotropy, and the linear elastic TPU model did not reproduce large-strain nonlinear behavior. The numerical results were therefore interpreted as comparative structural screening predictions for the assigned assumptions. The mesh was selected as optimal to achieve converged results and a lower computational cost. A refined mesh was employed in regions expected to experience higher stresses, specifically at edges, lattices, finger support, and sharp geometric transitions. These regions are more prone to stress concentrations and higher contact pressure; therefore, smaller meshes were employed to capture the mechanical response. The distal end of the forearm was fixed to replicate a boundary condition where the orthosis movement is supposed to be fixed (Figure 2b). This will also prevent rigid body motion and provide comparative stabilization supported by the arm during use. The orthosis was subsequently fixed at the finger ends to replicate the tight fit on the fingers and load transfer pathways observed during wear in the simulation. The arm geometry was used only to design the orthosis and was not included as a deformable anatomical body in the structural FEA. The distal orthosis region, end of the forearm, and the region before the start of the fingers were fixed, while the finger regions were not fully included in the TPU simulation to check the maximum or allowable movement of the fingers. No arm–orthosis contact pressure was computed in these results. Loading was implemented in four incremental time steps, helping to smooth convergence for the solver and simulating the increasing force of an orthosis as it begins to bear load (e.g., initial seating, progressive engagement, and final stable condition). Thereafter, the orthosis was investigated under these incremental loading conditions in accordance with the methodology described in the prior study [10], and results such as stress distribution within the orthosis patterned over it were analyzed. The FE data were utilized not as a final objective but as a design input instrument. The honeycomblike lattice regions were assigned to a refined mesh. The stress distribution obtained from the initial FEA was used to identify mechanically critical regions and guide localized geometric modifications. Rather than applying uniform thickness or arbitrary weight reduction, material distribution was adjusted according to the predicted mechanical response to achieve weight reduction while preserving continuous load paths. Additionally, sensitive areas for humans, recognized as potential discomfort zones, were also modified through material removal (as shown in the designated area in Figure 2e to reduce pressure on the median nerve in the carpal tunnel region). The proposed design (Figure 2d) was used to integrate all the objectives identified during the FEA-guided development process. Lattice configurations were refined in consistently low-stress areas to save mass while preserving structural integrity for lightweighting purposes, as stress path mapping can be used to identify regions that contribute less to global structural stiffness and therefore represent candidates for material removal while preserving primary load transfer pathways [23]. The arrangement of these structures on the hand and forearm panels facilitated improved ventilation for comfort during prolonged use. Finally, the weight of the initial orthosis was reduced from 258.27 g to 205.36 g, making it more comfortable for the user and reducing waste. Moreover, pressure distribution was improved through localized material removal and a specific wrist clearance zone, reducing direct compression on sensitive regions like the carpal tunnel.

**Figure 3:**
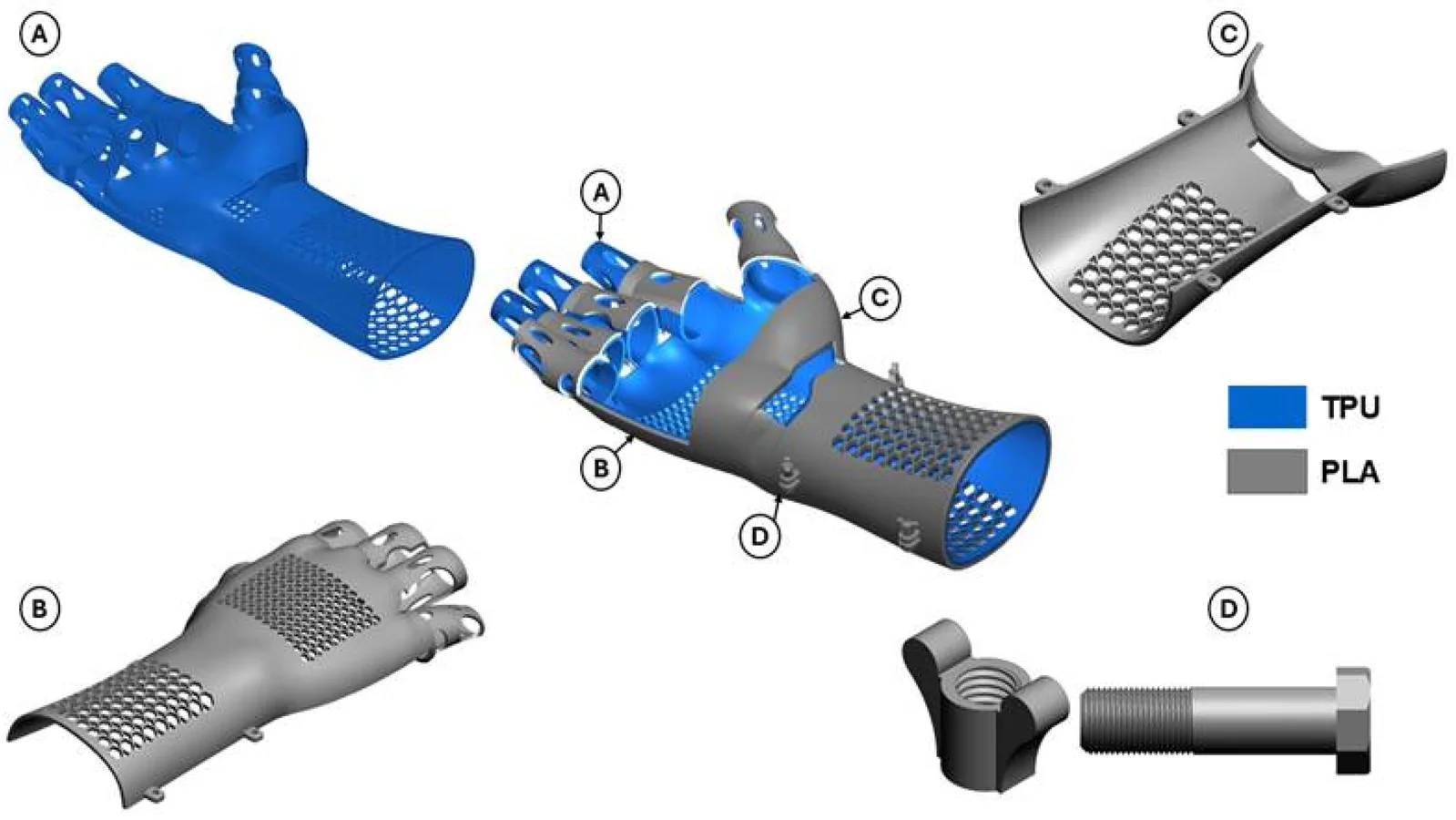
Assembly model of the multi-material wrist-to-finger orthosis geometry: (**A**) inner TPU part, (**B**) upper PLA part, (**C**) lower PLA part, (**D**) bolt and wingnut.

Stress contours from the baseline PLA shell model were inspected to identify the principal load paths and regions that remained under comparatively low stress in the selected screening cases (Figure 1, Step V), performed using ANSYS 2024R2, and further design improvements were made. As illustrated in Figure 3, material was selectively removed from low-stress regions to reduce the weight of the printed component, and ventilation openings were incorporated to enhance airflow and heat dissipation. This was expected to help minimize sweat accumulation and improve comfort. These modifications also reduced print time and material consumption, improving manufacturing efficiency without compromising usability or structural integrity. The previous literature distinguishes formal topology optimization from manually designed perforation strategies [12].

PLA and TPU were intentionally paired to balance rigid support with softer contact with the skin of the human body. PLA was chosen for its printing reliability and shape retention after fabrication. Its relatively high stiffness helps the orthosis act like a frame, maintaining wrist alignment, resisting unwanted bending, and providing the immobilization needed while allowing material removal in regions associated with carpal tunnel pressure relief. In practice, PLA can also be used to produce fine features such as vents, lattice regions, and finger support geometry, which are useful when the design includes thin walls and detailed openings. TPU was selected for the inner liner to improve comfort where the device touches the body. Unlike a rigid shell, TPU can be flexible and conform to the natural contours of the forearm, wrist, and fingers. This flexibility is especially important around sensitive regions where solid materials can create localized pressure points, as well as around the unique features of the human hand. The TPU liner also acts as a cushion, which helps spread contact forces over a larger area. This lowers sharp pressure peaks at edges, perforations, and finger interfaces. It also adds a small amount of movement, which can improve wear tolerance during day-to-day movement and helps accommodate minor swelling or volume changes without making the orthosis feel overly tight. Together, the materials work as a layered system. Prior wrist–hand orthosis studies have successfully used other material systems, including nylon [12], ABS-TPU combinations, TPU/PLA-based strategies [24], and PET-G [25]. PLA provides the primary structural stiffness and shape retention, whereas TPU provides a softer and more conformable skin contact interface (not quantified in this study) that may improve fit and perceived comfort without eliminating the stabilizing function of the rigid component. In addition, the mechanical properties of FFF PLA and TPU depend on layer orientation, raster, temperature, layer thickness, porosity, and other process variables [26,27,28].

### 2.3. Additive Manufacturing Process

**Figure 4:**
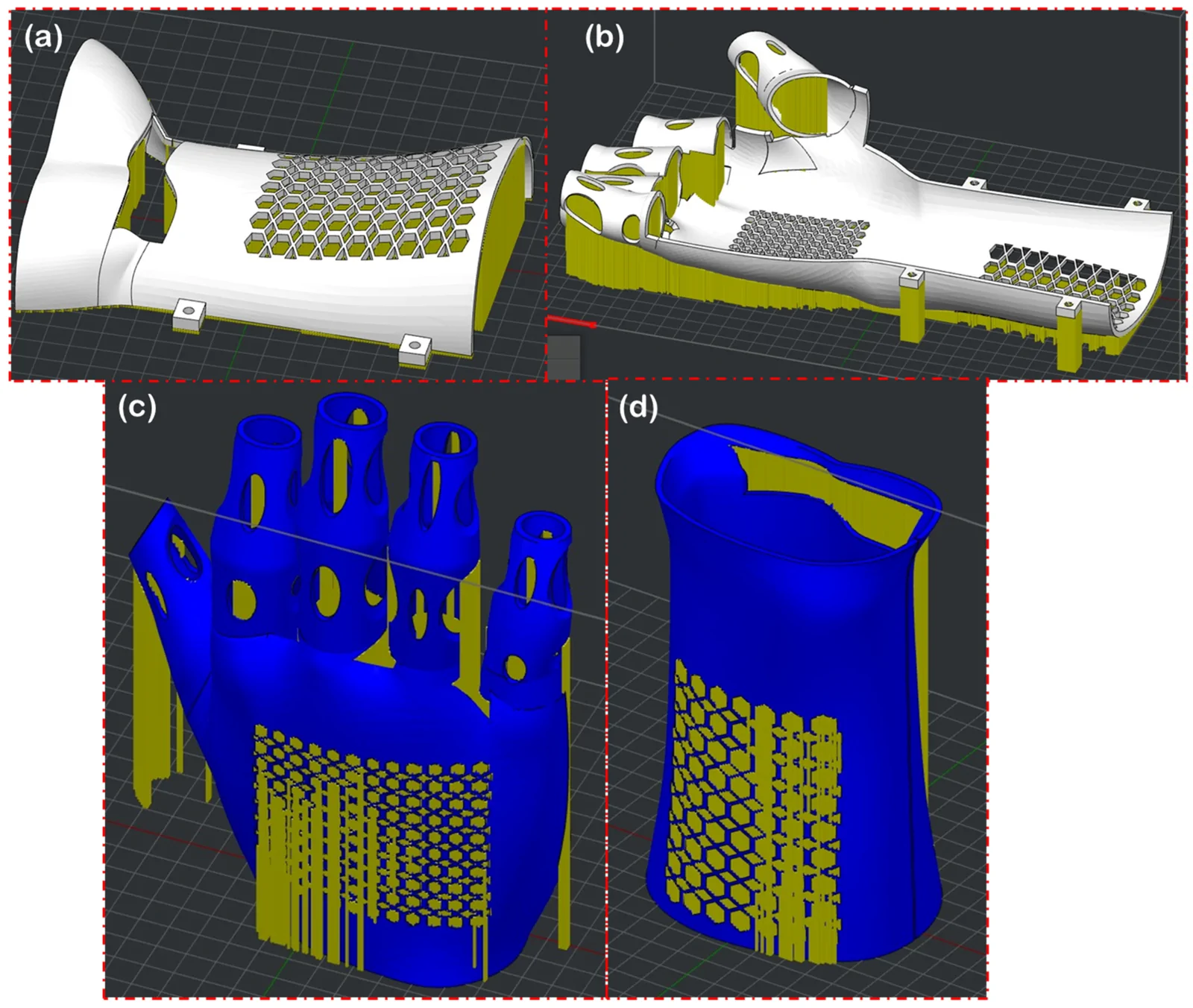
(**a**,**b**) The printing orientation and support strategy for the multi-material fabrication of the PLA parts of the wrist-to-finger orthosis. (**c**,**d**) The TPU parts were printed in an orientation selected for consistent fit and reduced post-processing requirements. Support structures are shown in yellow in all images.

The orthosis was fabricated using a multi-material FFF technique, incorporating a rigid PLA shell and a flexible TPU inner liner. Both the top and bottom PLA parts were produced separately and subsequently assembled with nuts and bolts to allow easy wearing and removal. For TPU, the hand and forearm were printed separately and then combined to form the final product. Boundaries in material change were designed with smooth geometric transitions rather than abrupt increments. This reduces stress concentration and facilitates a smooth transition between rigid (PLA) and flexible (TPU) materials. The printing was executed in the orientation depicted in Figure 4, which was selected to reduce the amount of support material used. This orientation enhances surface quality in areas of contact, reduces post-processing requirements, and helps in maintaining the accuracy of features such as lattice thickness and finger support geometry.

**Figure 5:**
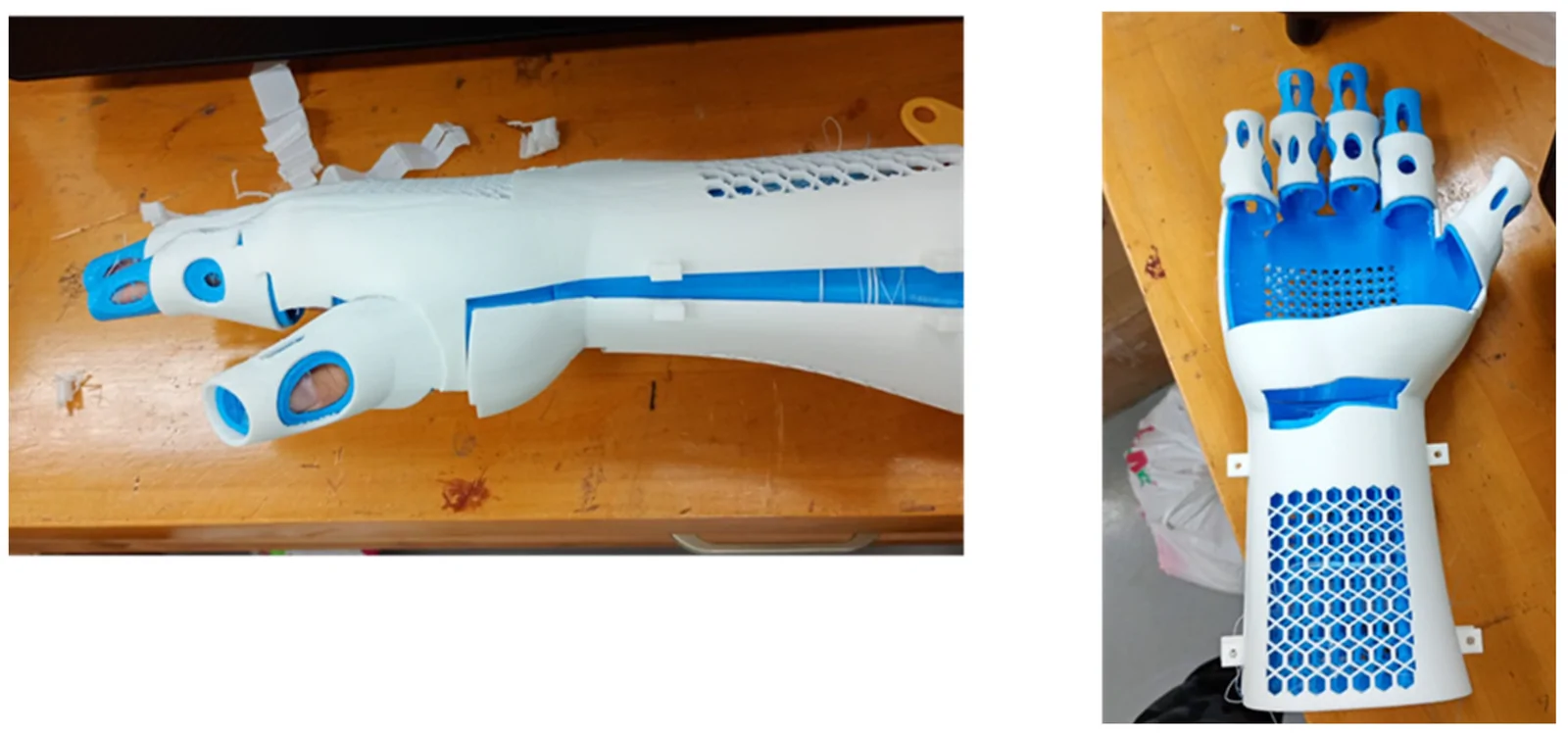
Prototype photos of the multi-material FFF-fabricatedorthosis (PLA, white; TPU, blue).

The Raise3D E2 3Dprinter (Raise3D, Shanghai, China) was used for the fabrication of the multi-material orthosis. Ideamaker was used to slice the 3D model, as recommended by the printer manufacturer. For the PLA, the nozzle temperature was set to 210 °C, with a heated bed temperature of 60 °C. The shell was printed with 100% infill using a rectilinear infill pattern. For the TPU liner, the nozzle temperature was increased to 220 °C, while the remaining printing settings were kept consistent with those of the PLA (including the 60 °C bed temperature, 100% infill, and rectilinear infill strategy). Maintaining comparable infill and baseline parameters helped promote uniform build quality, while the higher TPU nozzle temperature supported stable extrusion and improved interlayer cohesion in the flexible regions [27]. Figure 5 shows the fabricated multi-material 3D-printed orthosis.

### 2.4. Mechanical Testing of the Fabricated Orthosis

**Figure 6:**
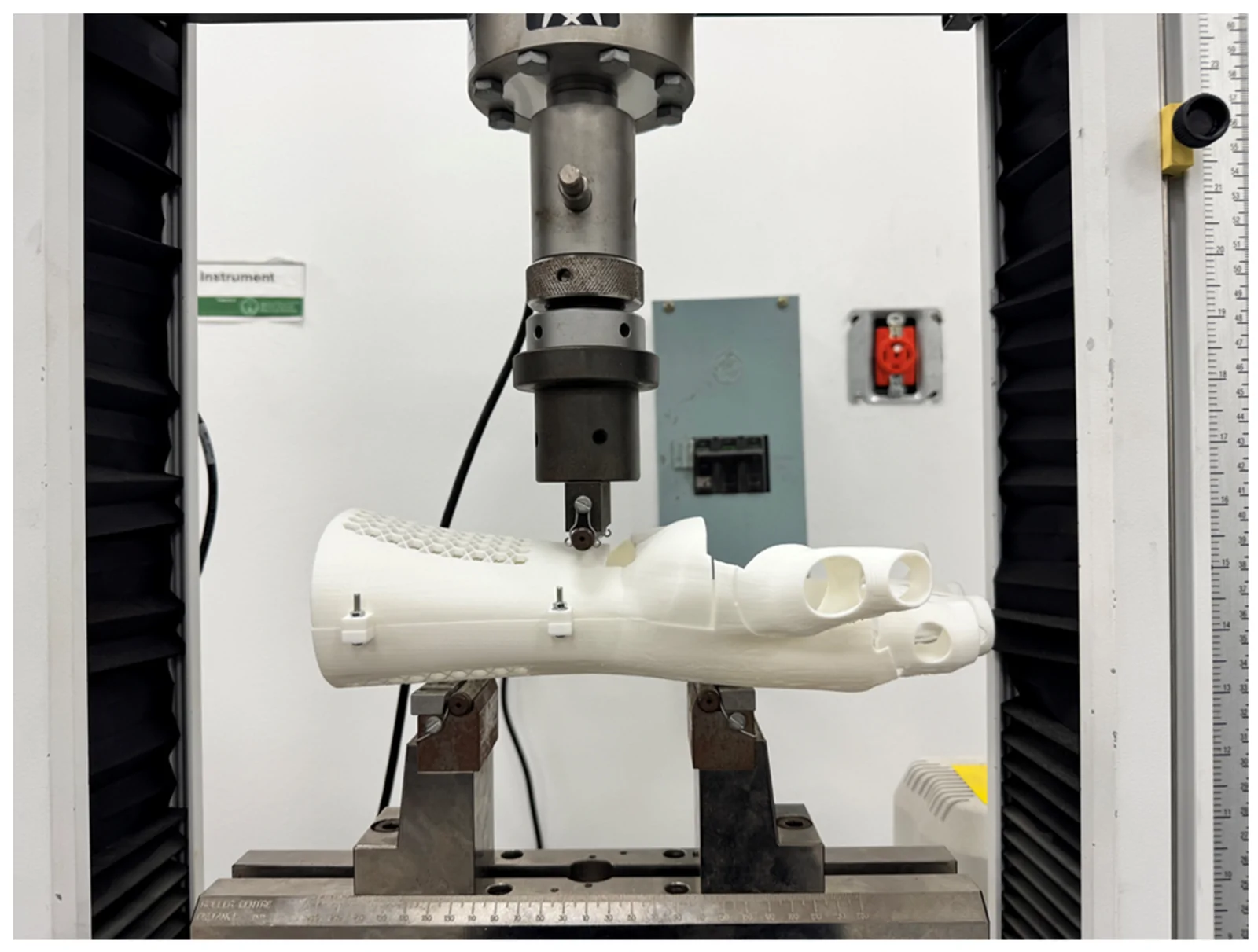
Flexural testing setup of the additively manufactured wrist orthosis using a Universal Testing Machine (UTM).

A flexural test was conducted, as shown in Figure 6, to evaluate the mechanical behavior of the multi-material additively manufactured orthosis under loading conditions. The test was performed using an Instron 3367 (Instron, Norwood, MA, USA) Universal Testing Machine(UTM) equipped with a 30 kN load cell. During testing, the upper loading anvil was positioned near the open region around the wrist, as shown in Figure 6, which represents a potential stress concentration zone in the orthosis design. Testing was conducted under displacement control at 2 mm/min to a maximum crosshead displacement of 40 mm. This location was selected because structural failure is most likely to initiate at geometric discontinuities such as the wrist opening and the joint interfaces. Applying the load at this critical region allowed assessment of the orthosis’ structural integrity and its ability to withstand bending stresses representative of real-world usage conditions [29]. Published orthosis studies use similar device-level test configurations to characterize material and structural response [12,25]. Because only one independently fabricated prototype was tested, the force–displacement response is reported descriptively, and no mean, standard deviation, or inference about specimen-to-specimen reproducibility is provided.

## 3. Results

### 3.1. Orthosis Fitting and Preliminary Evaluation

The final orthosis design incorporated ventilation features and lightweight perforations across both the hand and forearm panels. Ventilation openings can increase the open surface area and were incorporated to provide potential airflow pathways. A volar wrist clearance zone was integrated into the design to prevent direct compression over the carpal tunnel region, thereby improving user comfort and minimizing the risk of pressure-related complications. In addition, modular finger interfaces were implemented to facilitate controlled finger positioning, while access windows were incorporated to allow periodic skin inspection without removing the device. The fabricated prototype successfully demonstrates the implementation of the PLA-TPU material zoning strategy, where rigid PLA provides the primary structural support, and TPU is strategically positioned along the inner contact surfaces and finger interfaces to improve comfort and flexibility.

**Figure 7:**
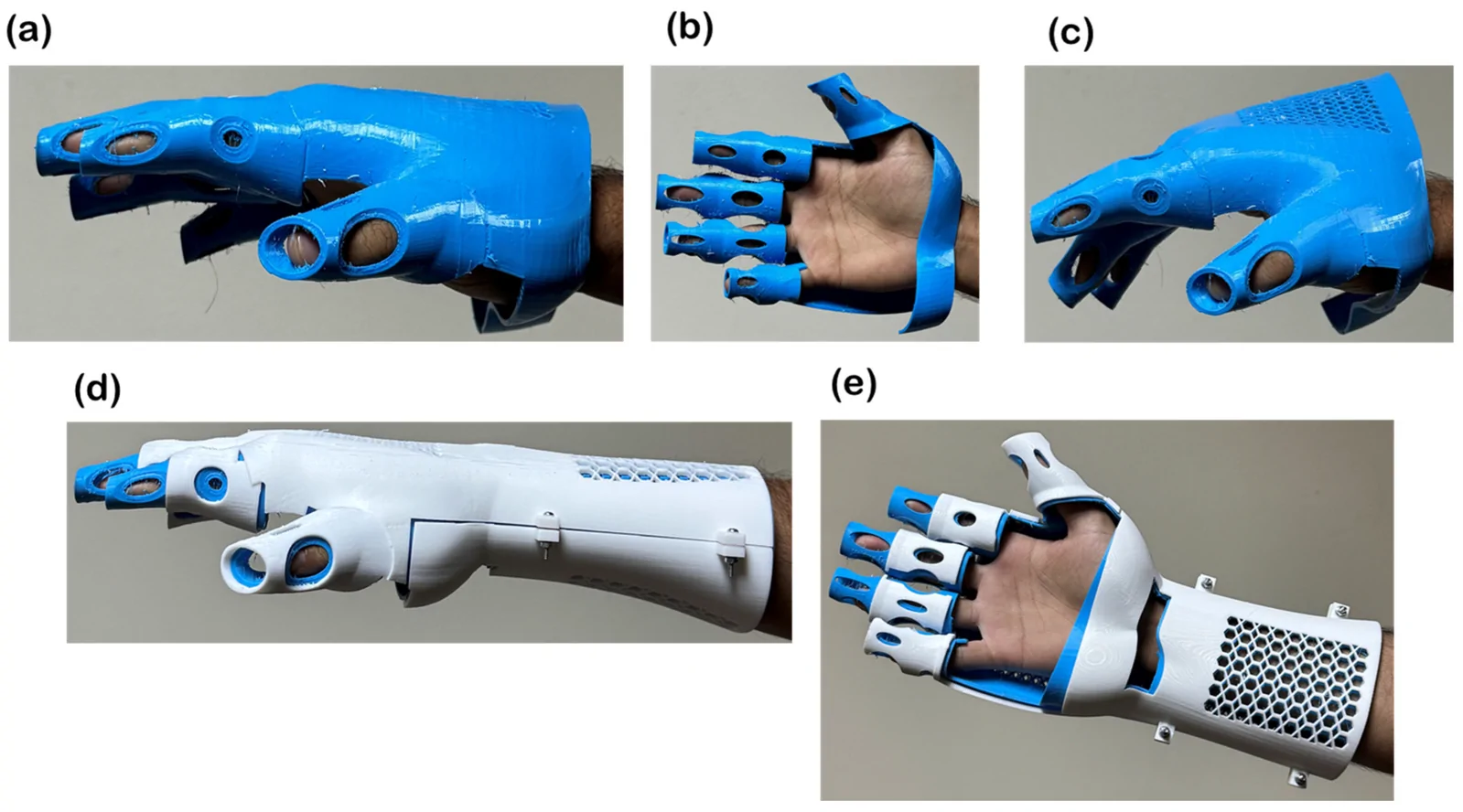
User fitting and evaluation of the additively manufactured PLA-TPU wrist orthosis. (**a**) Top view of the TPU inner interface fitted on the hand for initial comfort assessment. (**b**) Palmar view showing the TPU finger interfaces and wrist strap during preliminary fitting. (**c**) Evaluation of hand mobility while wearing the TPU component. (**d**) Side view of the fully assembled PLA-TPU orthosis fitted on the user’s hand. (**e**) Palmar view of the complete orthosis demonstrating the modular finger interfaces, forearm ventilation lattice, and overall ergonomic fit. No formal comfort, usability, pressure, or clinical assessment was performed.

Following fabrication, a preliminary fitting evaluation was conducted to assess user comfort and functionality. Initially, only the TPU component was tested to evaluate its fit and flexibility (Figure 7a,b). The user was also asked to perform basic hand movements to assess comfort and mobility, as illustrated in Figure 7c. Subsequently, the complete orthosis assembly, consisting of both the PLA and TPU components, was fitted to the user’s hand, as shown in Figure 7d,e. The preliminary observation indicated that the user’s hand rested comfortably within the PLA-TPU orthosis, demonstrating adequate fit and ergonomic support. Although wing nuts would have provided a more convenient fastening solution for assembly and adjustment, Phillips-head nuts and bolts were used instead due to the unavailability of wing nuts at the time of fabrication. The orthosis could be positioned on the hand and removed without apparent geometric interference, and the finger supports remained aligned with the corresponding digits.

### 3.2. FEA-Guided Structural Refinement and Mass Reduction

**Figure 8:**
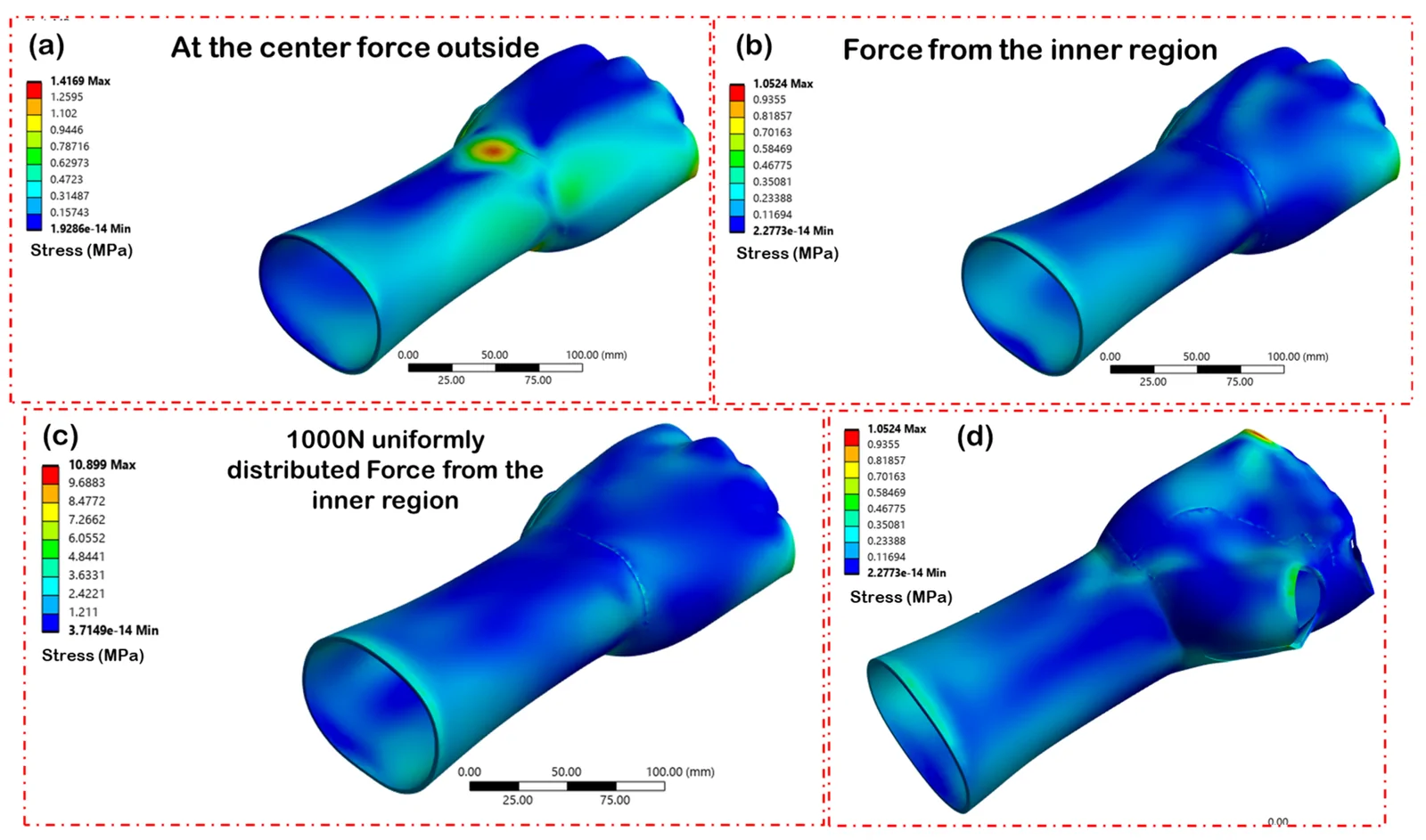
(**a**) External point load applied in the wrist region (1000 N) while the orthosis is positioned on the hand, highlighting localized peak stress regions. (**b**) Incremental loading applied on the inner (contact) surface using a time-step force representing progressive engagement of the orthosis with the arm. (**c**) Uniformly distributed 1000 N load applied in the inner region to evaluate global stress response and distinguish low-stress areas suitable for lattice expansion. (**d**) View of the stress from the backside of the orthosis to verify critical regions across the full geometry.

The optimization process began by performing an FEA on the full orthosis assembly (without fingers), and the resulting stress distributions are summarized in Figure 8. This first simulation was used as an example to show how loads travel through the orthosis, where the material is needed, and from which places we could safely remove it to reduce weight and increase ventilation without sacrificing stability. In Figure 8a, a 1000 N load is applied at the wrist region, acting externally on the orthosis while it is positioned over the patient’s hand. The selection of this load should not be considered representative of routine wrist or hand loading. Published computational investigations of customized wrist–hand orthosis have used substantially lower loads for functional loading scenarios, including 50 N and 150 N (Section 3.3), while experimental studies have reported considerably different failure loads depending on orthosis geometry, material, and additive manufacturing conditions [30]. In the present study, 1000 N was therefore deliberately treated as a conservative structural overload condition. Its purpose was to identify potential structural vulnerabilities and evaluate whether the optimized architecture retained a sufficient safety margin under a severe external load, rather than to imply that such a force is routinely experienced by the user [12]. The stress visualization from this step identified the primary load paths in the wrist and forearm sections and revealed where peak stresses are likely to occur during use. In Figure 8b, loading is applied from the inner (contact) side of the orthosis using four different time-step processes with different magnitudes [10]. This staged approach was used to improve numerical stability and, more importantly, to capture how stress patterns evolve as the orthosis first rests on the arm and begins to carry a load. Together, these simulations allow clear identification of high-stress regions that must be preserved or locally reinforced and low-stress regions where material removal is unlikely to compromise mechanical function.

The optimization simulations were conducted only for the PLA, as it forms the primary load-bearing structure in the design, while the TPU liner is intended mainly for comfort and pressure distribution at the skin interface rather than structural support. In other words, TPU improves wearability, but it is not relied on to carry the main structural loads, so the stress-driven design decisions were based on the PLA response. Finally, Figure 8c,d present the stress results viewed from the top and bottom of the orthosis. These views were examined under higher-loading conditions to ensure that material removal decisions were not based on a single stress visualization and that critical load paths were preserved across the entire geometry. Using stress maps from these multiple views, the lattice pattern was refined, and material was removed from consistently low-stress regions to increase ventilation and reduce weight. At the same time, high-stress regions were retained to maintain performance. This iterative, stress-guided refinement is in line with the overall clinical design goals, which are to lower pressure in the carpal tunnel, make the orthosis more breathable, and make it lighter without losing structural stability.

### 3.3. Stress Generated and Deformation of the Orthosis Under Different Loading Conditions

**Figure 9:**
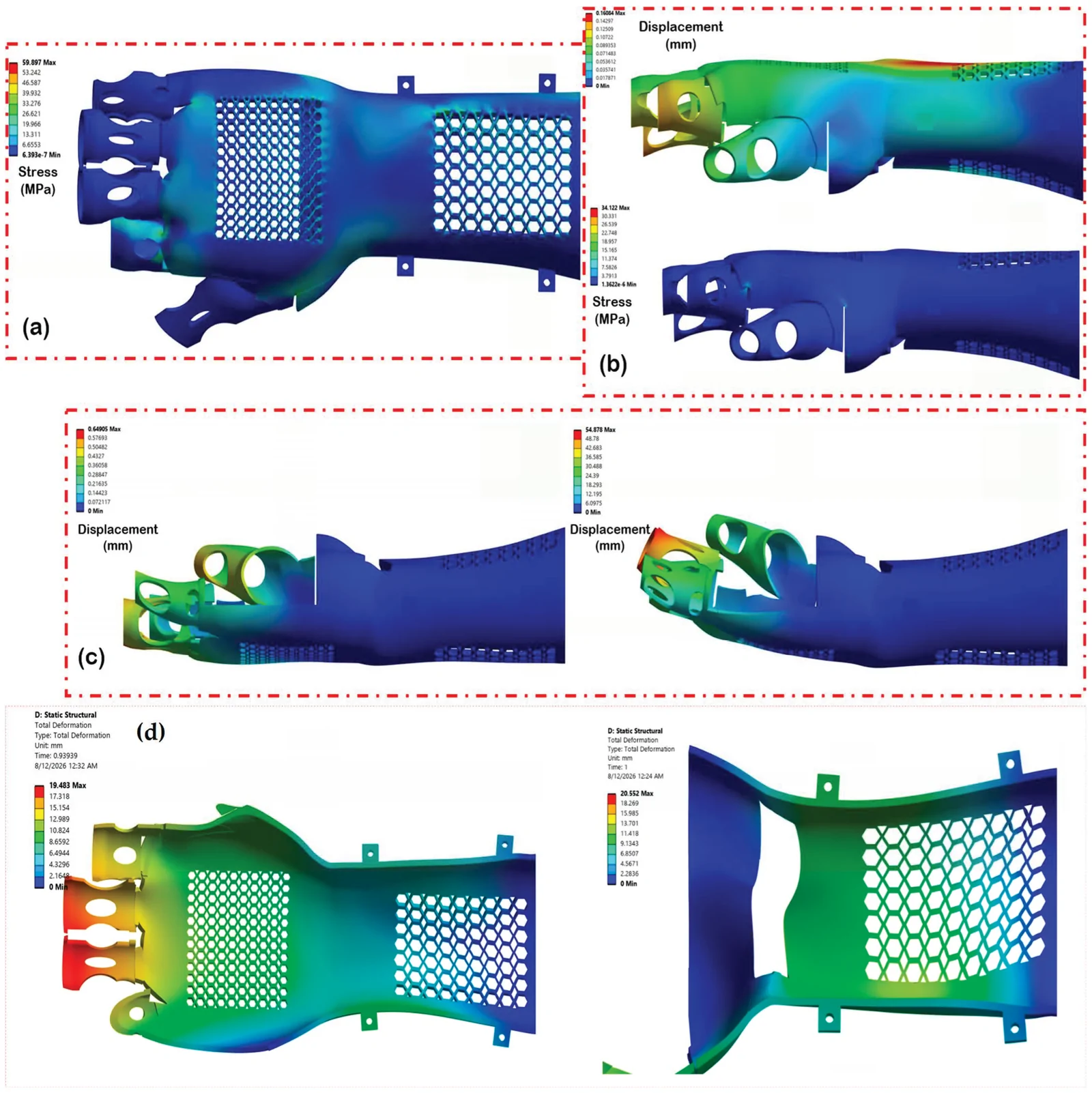
(**a**) Stress distribution (in MPa) in the PLA with lightweight lattice regions, highlighting the primary load paths and the locations of peak stress around structural transitions and finger support features. (**b**) Side-view results illustrating the overall deformation behavior: displacement field (mm, top) and the corresponding stress field (MPa, bottom) under the applied loading and boundary conditions. (**c**) Displacement contour (mm) showing how motion is concentrated near the distal finger support region while the forearm section remains stationary. The side view of the displacement confirms that the highest deformations occur at the finger when the material is TPU-only. (**d**) The prosthetic model split down the midline to reveal total deformation (mm) on both halves under the same loading conditions, with the left half showing deformation across the full arm and hand assembly (max ≈ 19.48 mm, concentrated at the finger and distal support region) and the right half showing the corresponding lattice panel section in isolation (max ≈ 20.55 mm), confirming that deformation is consistently concentrated toward the distal/finger end while the proximal forearm section remains largely undeformed.

The prediction of the stress distribution in the PLA after introducing the lightweight lattice regions is shown in Figure 9a at 1000 N. The contour plot clearly shows the main load transfer routes through the orthosis, with stress concentrating at expected transition locations, such as around changes in thickness, the edges of the lattices, and the junctions between the forearm shell and the finger support features. These spots were used to confirm that material removal did not affect critical structural integrity and that the remaining framework continued to carry the applied loads effectively. The side-view deformation response of the orthosis under the applied boundary conditions, presented in Figure 9b, shows the displacement (top) and the corresponding stress (bottom). Viewing both results together helps interpret how the orthosis behaves under the loading conditions, and regions with greater displacement indicate where flexibility is highest, while the stress plot highlights where the structure is most engaged in resisting the load. The stress analysis was also performed at 150 N force to see how the orthosis would behave at a relatively lower load. The value of the equivalent von Mises stress was 16.718 MPa. Overall, the forearm segment remained comparatively stiff and stable, while the finger support and upper regions showed a deformation of 0.16 mm, which is consistent with the design intent of maintaining the rigidity of the outer frame. For the TPU inner region, the maximum displacement was also evaluated when a small force of 10 N was applied to the end of the fingers to assess their movement, as shown in Figure 9c, to check whether finger motions would be possible with this small amount of force. The displacement contour emphasizes that deformation was primarily concentrated near the finger support region, while the forearm and wrist sections exhibited limited movement. This pattern is desirable because it indicates that the orthosis maintains stability while accommodating small deflections at the finger interfaces. The side-view displacement response also shows that the greatest deformation occurred in the finger support region. This result is because TPU is more flexible, and this observation supports the multi-material design strategy: PLA provides the structural backbone to control motion, while TPU is better suited for comfort-driven interfaces where localized flexibility and pressure smoothing are required. Figure 9d presents the model split down the midline to allow closer inspection of the total deformation on each half of the structure under the applied loading conditions. The left half, showing the complete arm-and-hand assembly, reached a maximum deformation of approximately 19.48 mm, with the highest values concentrated in the finger support and distal regions and deformation decreasing progressively toward the proximal forearm section, which remained largely stable. The right half, isolating the lattice panel section, exhibited a maximum deformation of approximately 20.55 mm, also concentrated near the finger support end of the panel. These results are consistent with the displacement trends observed in Figure 9c, confirming that the lightweight lattice regions do not introduce localized weak points but instead maintain a deformation pattern similar to the solid sections, with displacement governed primarily by proximity to the distal loading and support features rather than by the lattice geometry itself.

### 3.4. Variation of Stress in Different Regions of the Orthosis

**Figure 10:**
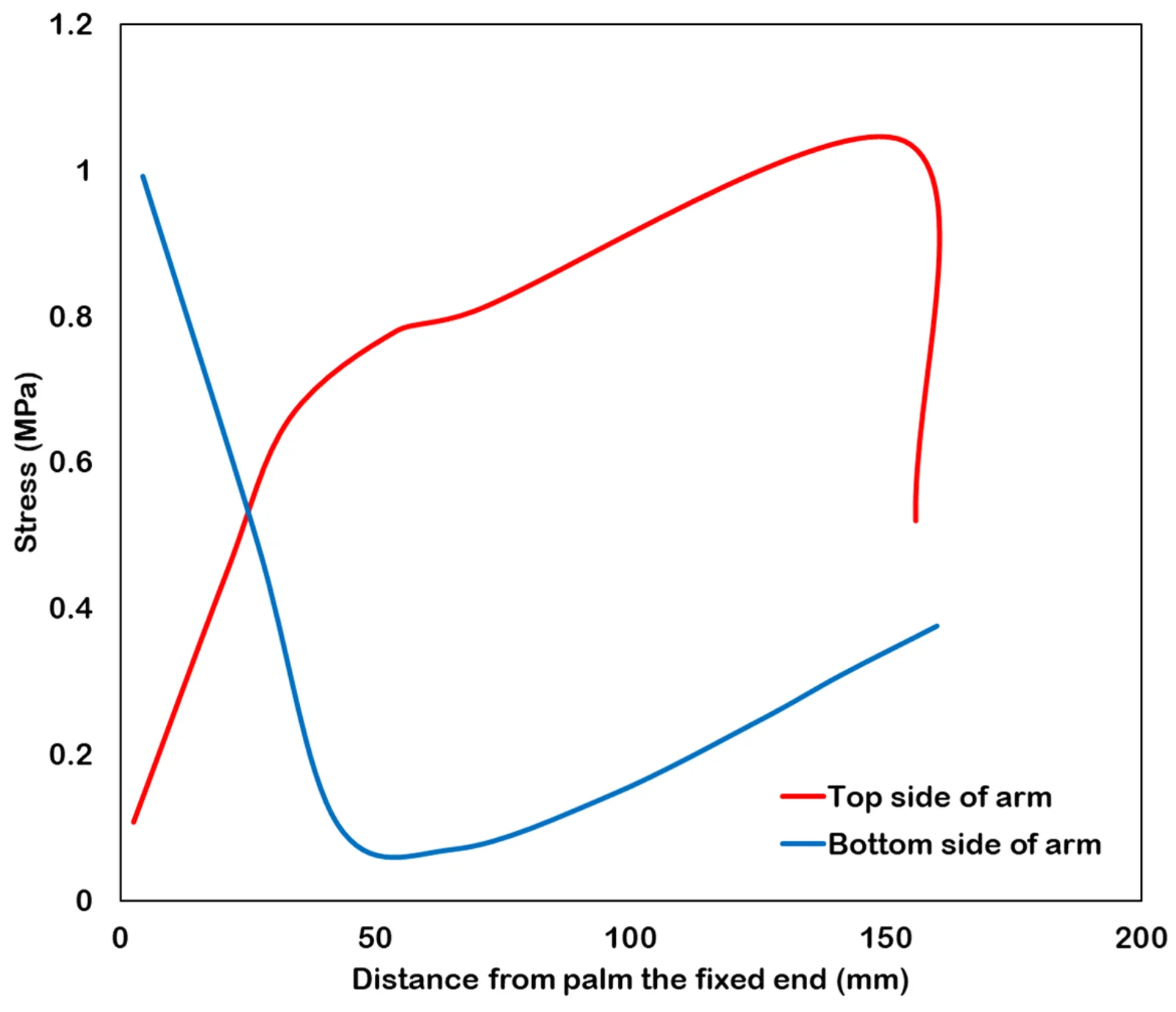
Stress variation along the orthosis length on the top and bottom sides at varying distances from the palm to the fixed end.

After examining the stress contour plots in the preceding sections, it was also important to evaluate how stress varied along the length of the orthosis, as presented in Figure 10. Rather than relying solely on stress contours, it was also quantified using a probe-based approach, in which discrete node probes were placed at representative locations on both the top and bottom surfaces of the orthosis. This provided a clear, numerical description of the stress distribution, allowing the stress distribution to be directly compared between regions and surfaces.

**Figure 11:**
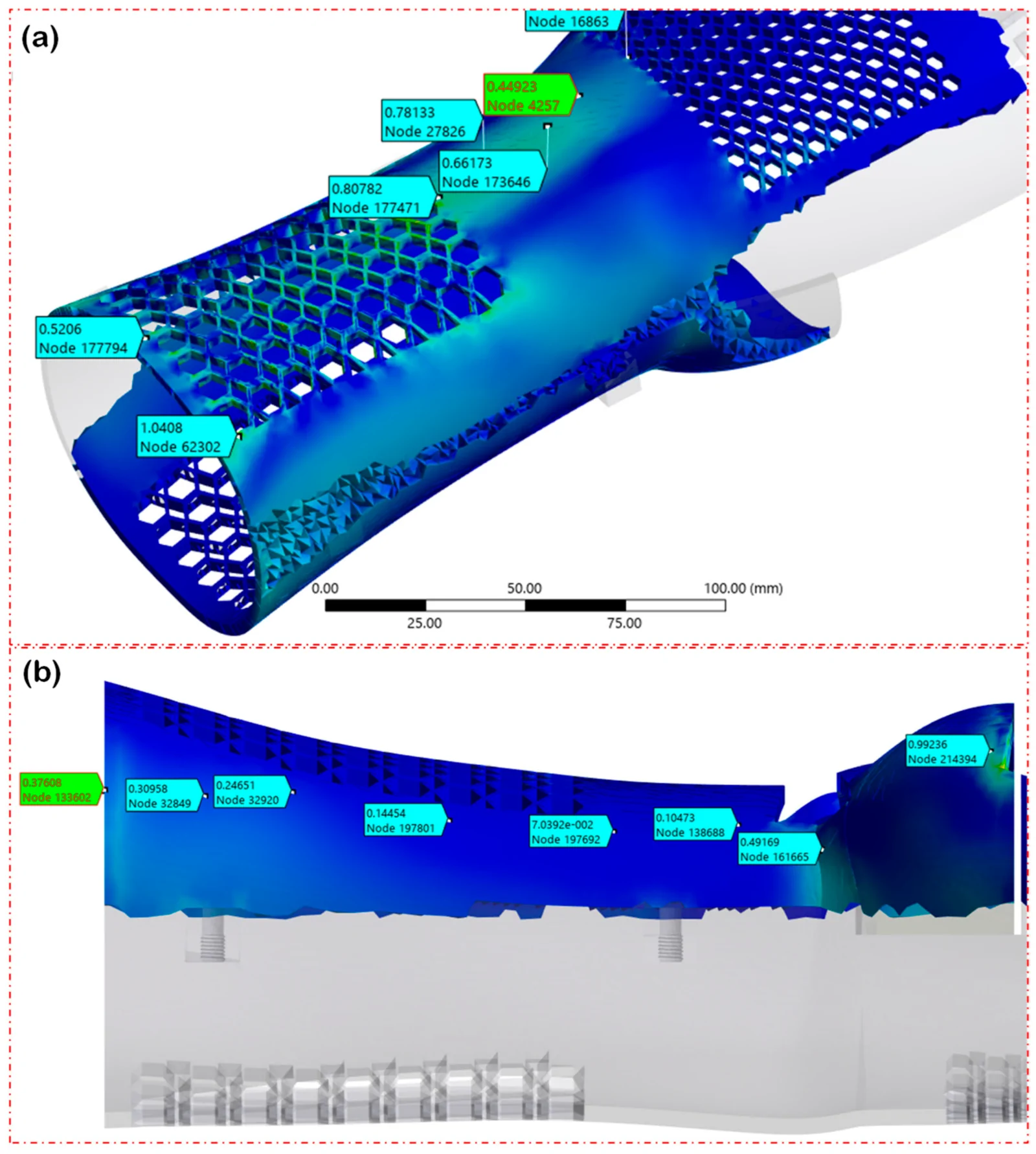
(**a**) The stress probes distributed over the top surface of the orthosis, including regions near the lattice and geometric transitions, and (**b**) the corresponding probes on the bottom surface, capturing stresses along the forearm span and toward the carpal tunnel region.

The stress analysis for this part was performed at 20 N, which is greater than that performed for TPU, in an attempt to replicate the force that a person can apply if he/she slightly moves his/her fingers. However, this force was not measured or clinically proven but was approximated from another study [31]. Probe locations were selected to capture both likely high- and low-stress regions. In particular, probes were placed near features that commonly generate stress concentrations, such as the edges of lattice boundaries, geometric transitions, and thickness changes, as well as the wrist region, where load transfer and carpal tunneling are typically most pronounced. Additional probes were distributed along the forearm section, which was expected to experience comparatively lower stresses and therefore represents the most promising area for weight reduction through expanded ventilation patterns or material removal. This approach was used to ensure that stress comparisons were based on the predefined stress sampling strategy that was used to quantify the spatial variation in stress along the orthosis. The extracted probe values (shown as labeled stress values in Figure 11) provided a numerical stress profile along the orthosis. This approach makes it possible to identify zones that consistently remain under low stress, supporting decisions to safely reduce material in those areas to reduce weight and improve ventilation. At the same time, probe data highlight regions with elevated stresses that should be preserved or locally reinforced to ensure structural continuity, maintain immobilization performance, and prevent unintended deformation underloading. Overall, the probe-based stress evaluation complements the contour visualization by providing an objective basis for targeted design refinement.

### 3.5. Flexural Testing of Multi-Material Orthosis

**Figure 12:**
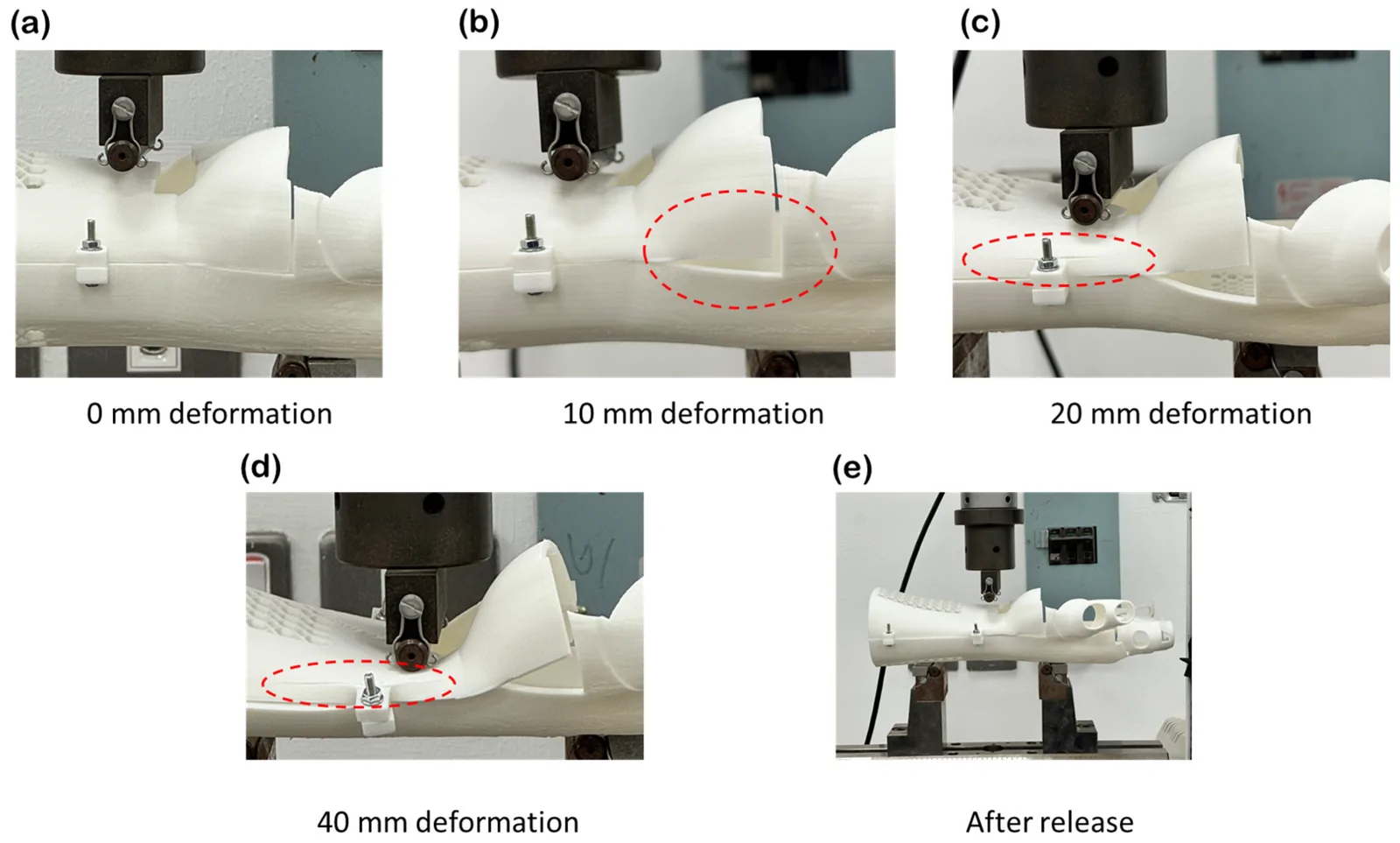
Flexural testing of the additively manufactured wrist orthosis under three-point bending. (**a**) Initial undeformed state before loading (0 mm displacement). (**b**) Deformation at 10 mm displacement showing the onset of bending and lifting of the upper section. (**c**) Deformation at 20 mm displacement with crack initiation near the joint region, highlighted by the dashed circle. (**d**) Continued crack propagation at 40 mm displacement under increased bending load. (**e**) Recovery after unloading.

Flexural testing was conducted using a Universal Testing Machine (UTM) equipped with a 30 kN load cell and a three-point bending fixture consisting of two lower supports and a rounded upper loading nose. The tests were performed under displacement-controlled conditions at a constant crosshead speed of 2 mm/min to simulate quasi-static loading. The three-point flexural configuration was selected as a device-level structural screening test representative of an external impact or accidental knock against the orthosis rather than the physiological outward loading exerted by the limb during normal wear. This test isolates the shell’s resistance to bending at its most geometrically discontinuous region (the wrist opening) and provides a conservative structural benchmark. It is not intended to reproduce interior contact pressure, which is instead addressed in the internal loading FEA case in Section 3.2 (Figure 8b). The crosshead displacement was progressively increased to a maximum of 40 mm, and the deformation state of the orthosis was documented at key intervals of 0, 10, 20, and 40 mm, as illustrated in Figure 12a–d. After reaching the maximum displacement, the specimen was unloaded to assess elastic recovery and identify any permanent deformation in the structure (Figure 12e).

**Figure 13:**
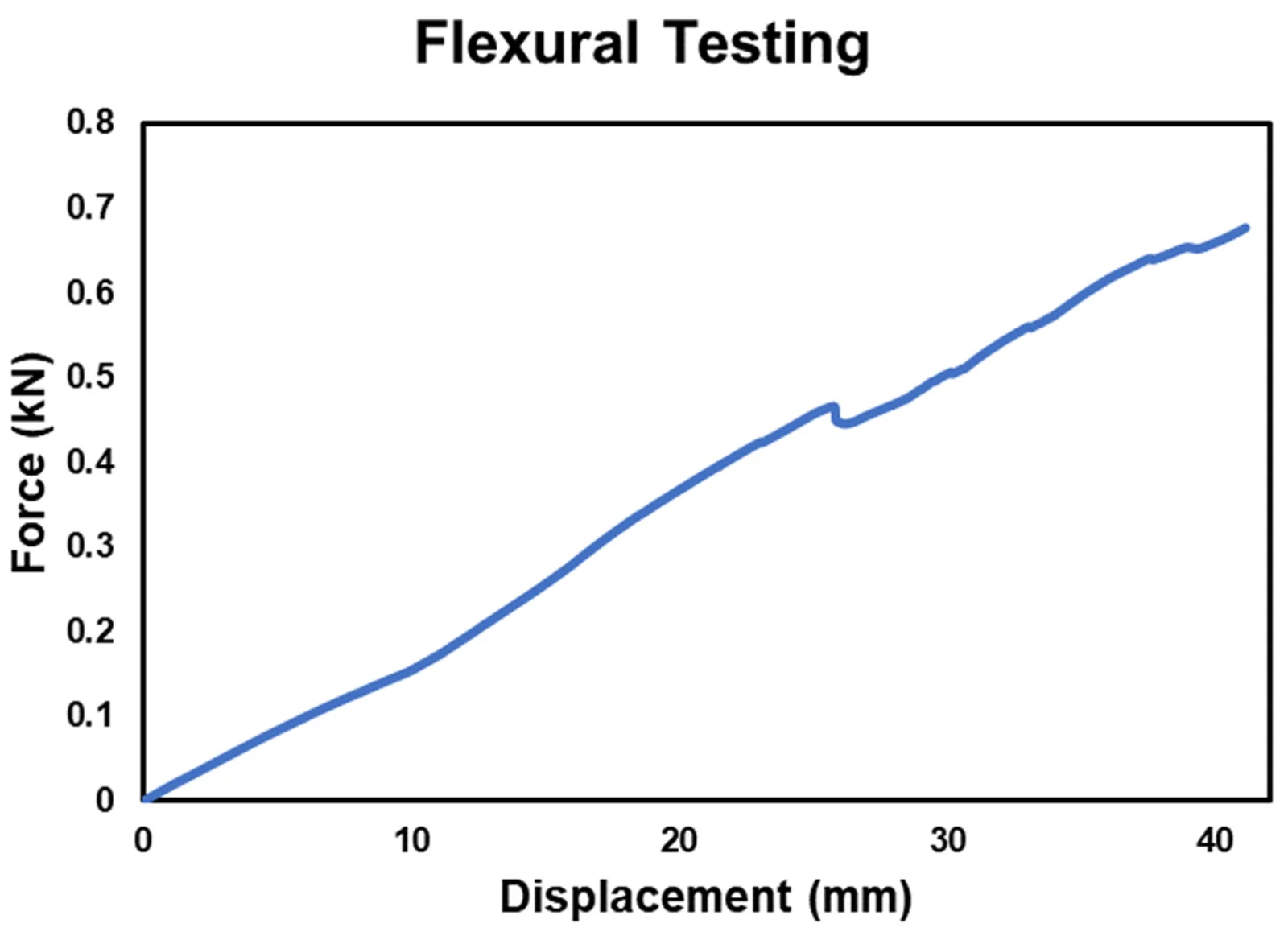
Force–displacement curve obtained from the three-point flexural testing of the additively manufactured wrist orthosis.

The force–displacement curve obtained from the flexural test (Figure 13) shows a gradual increase in load with increasing displacement, indicating a predominantly elastic response of the orthosis during the initial loading stage. From 0 to approximately 10 mm displacement, the force increases almost linearly, reaching about 0.15 kN. This region corresponds to the initial bending of the structure, where the upper section of the orthosis begins to lift from its resting position due to the applied load.

As the displacement increases from 10 mm to approximately 26 mm, the force continues to rise steadily, reaching nearly 0.4–0.45 kN. This indicates that the orthosis is effectively resisting the applied bending load while maintaining structural stiffness. At around 26 mm displacement, a small but noticeable drop in the force–displacement curve is observed. This drop corresponds to crack initiation near the joint region, which acts as a geometric discontinuity and a stress concentration point. The sudden reduction in force reflects the local loss of stiffness caused by the onset of damage in the structure.

Beyond 26 mm displacement, the force begins to increase again as the loading continues, reaching approximately 0.67 kN at the maximum displacement of 40 mm. This behavior indicates that, although a crack had initiated, the structure was still capable of carrying additional load, suggesting progressive crack propagation rather than catastrophic failure. Although a localized crack was observed near the stress concentration region, the global structure retained its overall geometry after unloading.

## 4. Limitations and Validation Requirements

Despite the promising results obtained in this study, several limitations should be acknowledged. First, the FEA did not incorporate a coupled anatomical contact model. Therefore, intra-tunnel pressure and detailed skin–orthosis contact pressures were not directly computed. The applied loading conditions were also designed as representative screening cases intended to identify general structural behavior rather than being calibrated to standardized clinical tasks or realistic strap forces experienced during daily use. The agreement between the numerical and experimental responses indicates that the FEA provides a reasonable approximation of the global elastic response of the fabricated orthosis under the laboratory loading configuration. However, the model should not be interpreted as predicting crack initiation or post-cracking behavior because the current formulation assumes elastic–plastic material behavior and does not explicitly represent damage evolution or fracture. Actual bending simulation as performed in the three-point bending test was not validated in the experimental study. Finally, this work focused primarily on engineering design and mechanical evaluation of the orthosis, and, therefore, clinical outcomes such as symptom improvement, user adherence, or nerve conduction changes were not assessed.

Future work will focus on addressing these limitations to further improve the design and clinical relevance of the orthosis. A more advanced coupled finite element contact model incorporating soft tissue representation, contact mechanics, and frictional interactions will be developed to quantitatively evaluate interface pressures, particularly in the carpal tunnel region. Experimental measurements using thin-film pressure sensors will also be conducted to validate interface pressure predictions and to study the effect of ventilation perforations on thermal comfort. In addition, more accurate material models will be established by performing tensile and bending coupon tests on specimens printed with the same PLA-TPU process parameters used for the orthosis, allowing calibration of nonlinear and anisotropic material behavior. Finally, a clinical feasibility study will be conducted to evaluate the orthosis in real-world use, focusing on user comfort, adherence, and potential improvements in patient symptoms.

## 5. Conclusions

This study presented the design, fabrication, and experimental evaluation of a customized proof-of-concept wrist orthosis manufactured using FFF with a dual-material PLA-TPU configuration. The use of stress-driven design optimization helped reduce weight and improve ventilation while keeping the important load-bearing regions intact. The addition of a volar wrist clearance and flexible TPU interfaces also aided in improving pressure distribution and overall user comfort. Mechanical testing showed that the orthosis had good stiffness and recovered much of its macroscopic shape after unloading. Although a small crack initiated near a stress concentration region, the structure was still able to carry additional load and maintain its overall performance at high stress levels.

Overall, the proposed design shows that combining FEA with multi-material FFF can be useful for developing a lightweight, comfortable, and mechanically stable orthosis. The design incorporates ventilation openings, adjustable fastening, inspection windows, and a volar clearance zone to address practical considerations associated with prolonged orthosis use. The effects of these features on circulation, tissue loading, thermal comfort, and long-term usability require dedicated experimental and clinical evaluation. In addition, the design can make daily life easier for patients by making the orthosis easier to clean and remove and by improving its water resistance. Future work may focus on optimizing joint geometry, evaluating cyclic and multidirectional loading, improving fastening mechanisms, and conducting comprehensive clinical evaluations to further validate the effectiveness of the orthosis in real-world rehabilitation applications.

## Data Availability

The original contributions presented in this study are included in the article material. Further inquiries can be directed to the corresponding author.
